# 6-Gingerol stabilized the p-VEGFR2/VE-cadherin/β-catenin/actin complex promotes microvessel normalization and suppresses tumor progression

**DOI:** 10.1186/s13046-019-1291-z

**Published:** 2019-07-02

**Authors:** Weilong Zhong, Wendong Yang, Yuan Qin, Wenguang Gu, Yinyin Xue, Yuanhao Tang, Hengwei Xu, Hongzhi Wang, Chao Zhang, Changhua Wang, Bo Sun, Yanrong Liu, Huijuan Liu, Honggang Zhou, Shuang Chen, Tao Sun, Cheng Yang

**Affiliations:** 1 State Key Laboratory of Medicinal Chemical Biology and College of Pharmacy, Nankai University, Tianjin, 300350 China; 2 Department of Gastroenterology and Hepatology, Tianjin Institute of Digestive Disease, Tianjin Medical University General Hospital, Tianjin, 300041 China; 3 Tianjin Key Laboratory of Early Druggability Evaluation of Innovative Drugs and Tianjin Key Laboratory of Molecular Drug Research, Tianjin International Joint Academy of Biomedicine, Tianjin, 300450 China

**Keywords:** Microvascular structural entropy, 6-gingerol, Tumor vessel normalization, Tumor microenvironment

## Abstract

**Background:**

Anti-angiogenic therapies demonstrate anti-tumor effects by decreasing blood supply to tumors and inhibiting tumor growth. However, anti-angiogenic therapy may leads to changes in tumor microenvironment and increased invasiveness of tumor cells, which in turn promotes distant metastasis and increased drug resistance.

**Methods:**

The CO-IP assays, N-STORM and cytoskeleton analysis were used to confirm the mechanism that p-VEGFR2/VE-cadherin/β-catenin/actin complex regulates vascular remodeling and improves the tumor microenvironment. 6-gingerol (6G), the major bioactive component in ginger, stabilized this complex by enhancing the binding of VEGFa to VEGFR2 with non-pathway dependent. Biacore, pull down and molecular docking were employed to confirm the interaction between 6G and VEGFR2 and enhancement of VEGFa binding to VEGFR2.

**Results:**

Here, we report that microvascular structural entropy (MSE) may be a prognostic factor in several tumor types and have potential as a biomarker in the clinic. 6G regulates the structural organization of the microvascular bed to decrease MSE via the p-VEGFR2/VE-cadherin/β-catenin/actin complex and inhibit tumor progression. 6G promotes the normalization of tumor vessels, improves the tumor microenvironment and decreases MSE, facilitating the delivery of chemotherapeutic agents into the tumor core and thereby reducing tumor growth and metastasis.

**Conclusions:**

This study demonstrated the importance of vascular normalization in tumor therapy and elucidated the mechanism of action of ginger, a medicinal compound that has been used in China since ancient times.

**Electronic supplementary material:**

The online version of this article (10.1186/s13046-019-1291-z) contains supplementary material, which is available to authorized users.

## Background

Microvessels deliver oxygen and nutrients and remove waste from the tumor microenvironment [1, 2]. Although solid tumors have numerous blood vessels, the function and structure of these vessels are usually abnormal, characterized by high permeability, the absence of pericytes and non-specific extravasation of blood components. Abnormal microvessels can result in a hypoxic and an acidic microenvironment and increase the interstitial fluid pressure (IFP), which can induce epithelial-mesenchymal transition (EMT) and resistance to radiation therapy and chemotherapy, and these changes can even increase metastasis [3, 4].

Microvascular structural entropy (MSE), a measure of the degree of disorder, is used to assess the structural organization of microvessels. MSE combines measurements of the size and distance between vessels. Furthermore, we used fractal dimension analysis to quantitatively assess vascular complexity and tortuosity [5–7]. Until now, there has been a lack of studies on the role of MSE in tumor pathology. A regular or ordered microvascular distribution improves the tumor microenvironment and decreases malignancy by promoting microvascular normalization [8–11].

Ginger has been used for thousands of years in China as an “interior-warming drug”. It is used in the “Sini Decoction” and “Decoction for Restoring Yang from Collapse” as an adjunctive drug. Ginger can alleviate gastrointestinal paralysis and spasms and improve dysmenorrhea and cold stress [12–14]. Despite being used for more than a millennium, the mechanism of action of ginger is still unclear. Being the major bioactive component in ginger, 6G has similar effects to those of ginger [15–17].

Anti-angiogenic therapies attempt to “block” vessels to starve tumor cells (TCs) by inhibiting angiogenesis and/or destroying existing vessels [18–21]. However, anti-angiogenic therapies that inhibit angiogenesis often have side effects, such as the deterioration of the tumor microenvironment, leading to tumor progression, increased EMT and drug resistance [22]. In this study, we report that 6G can regulate the normalization and order of tumor microvessels and further suppress tumor progression. In addition, 6G can also increase the integrity of the endothelial cell (EC) barrier and tight junctions between and around the periphery of ECs. 6G alters the vascular morphology from multiple branches with short intervals to minor branches with long intervals, resulting in the reconstruction of the tumor microvasculature network and reduction of MSE and fractal dimension. The results of proteomics analysis indicated that 6G directly targets and activates VEGFR2. 6G enhances the binding of VEGFa to VEGFR2 to promote VEGFR2 phosphorylation. Phosphorylated VEGFR2 (p-VEGFR2) recruits VE-cadherin (VE-cad)/β-catenin/actin to regulate F-actin cytoskeletal remodeling in ECs, altering vascular remodeling, thereby promoting the normalization of tumor microvessel networks and improving the tumor microenvironment. These changes can increase the cytotoxic effects of chemotherapy drugs and prevent cancer progression.

## Materials and methods

### Cell culture

Alexander hepatoma cell line PLC/PRF/5 and breast cancer cell line MCF-7 were maintained in RPMI 1640 supplemented with 10% FBS and 1% PS (penicillin and streptomycin) and under 37 °C and 5% CO_2_. The cells were purchased from KeyGen Biotech (Nanjing, China) and the company provides complete cell identification. The cells periodically authenticated by biomarkers detection of hepatocellular carcinoma, morphologic inspection, growth curve analysis, and mycoplasma testing, and not used beyond 10 passages.

### Microvascular structural entropy (MSE)

HE-stained sections and CD34-stained tissues were quantitatively assessed using a Nikon NIS-Elements analysis system (Nikon, Japan). A 40× apochromatic objective was used to capture the images. To enhance the contrast between microvessels and their lumens, each image was digitally processed. In each immunostained preparation, 5 random fields were selected, and MSE was calculated. The following structural entropy equation was used to calculate MSE:

Here, MSE represents microvascular structural entropy; the microvessel area is represented by a, and d is the distance between neighboring microvessels. In the calculation of MSE using the minimum spanning tree method as the basic procedure, each microvessel should be related to only one neighboring microvessel [23]. Δa represents the mean area of all microvessels in one calculation field; Δd represents the mean distance between all neighboring microvessels. The detailed parameters are explained in the study by Kovacs et al. [24] When both the area and distance between the nearest-neighboring microvessels were constant for all microvessels, the value of MSE was zero. A greater difference in the areas and distances of the microvessels results in a larger MSE.

### Immunofluorescence staining

Adherent cells at 80% confluence were treated with 0, 15 and 30 μM 6G or DMSO for 12 h. Then, the medium was removed, and the cells were washed three times with PBS. The cells were fixed with 3.7% paraformaldehyde for 20 min, washed three times with PBS, treated with 0.1% Triton X-100 and 5% BSA for 30 min and washed again with PBS. Next, phalloidin-tetramethylrhodamine (TRITC) (1:50) was added, and the cells were incubated for 30 min. Finally, the cells were stained with 4′,6-diamidino-2-phenylindole (DAPI) (Sigma, USA), mounted and viewed using a laser-scanning confocal A1 microscope (Nikon, Japan).

### Western blotting

The cells were lysed in RIPA buffer with protease inhibitor cocktail (Sigma). Proteins were quantified and separated on a 10% SDS-PAGE gel, transferred to PVDF membranes, and incubated with primary antibodies. Pierce detection reagents were used to visualize the horseradish peroxidase-labeled secondary antibodies. Western blot results were collected by automatic analysis system of electrophoresis gel imaging (GenoSens, Shanghai). The relative band intensity was quantified using ImageJ v1.37 software (U.S. National Institutes of Health, Bethesda, MD, USA).

### Co-immunoprecipitation (IP)

Lysates were prepared by incubating the cells on ice with cold TBS-Nonidet P-40 lysis buffer (20 mM Tris, pH 7.4, 150 mM NaCl, and 1% Nonidet P-40) in the presence of protease and phosphatase inhibitor cocktails (Roche) for 30 min at 4 °C. Then, the cell lysate was centrifuged at 12,000×g for 10 min at 4 °C. For immunoprecipitation, proteins (approximately 0.5 mg) were incubated with control or specific antibodies (3–5 μg) for 12 h at 4 °C under constant rotation; 30 μl of protein G magnetic beads (Invitrogen) was then added, and the mixture was further incubated for 2 h. The beads were washed with lysis buffer and then collected using magnetic stands at 4 °C. The proteins were eluted by boiling for 10 min with 2× SDS-PAGE loading buffer. Immune complexes were analyzed by SDS-PAGE using appropriate antibodies.

### Murine xenograft model

All mice were purchased from Charles River Laboratories (Beijing, China). Four-week-old female nude mice were maintained at a specific pathogen-free animal facility at Tianjin International Joint Academy of Biomedicine. The mice were randomly divided into 3 groups (*n* = 10 mice/group), and 1 × 10^7^ MCF-7 cells were subcutaneously injected into the axilla. When the volume of the tumor reached approximately 10 mm^3^, the mice were treated with 100 or 200 mg/kg of 6G or saline (as a control) for four weeks. All mice were sacrificed through carbon dioxide inhalation. Subsequently, the tumor, lung and liver tissues were fixed for analysis. All animal experiments were performed under approved protocols of the institutional animal use and care committee of Tianjin International Joint Academy of Biomedicine.

For the CDX model with PLC/PRF/5 cells, mice were randomly divided into six groups (eight mice each) and treated with saline, cisplatin, sorafenib, sorafenib/cisplatin, 6G or 6G/cisplatin for four weeks.

### Immunohistochemical (IHC) analysis

Tumor tissues were embedded in paraffin and cut into 4-μm thick sections. Following uplink dehydration, microwave antigen retrieval and blocking, the samples were incubated with the following primary antibodies overnight at 4 °C: rabbit polyclonal anti-HIFα (Affinity, 1:100 dilution), rabbit polyclonal anti-MMP2 (Zhongshan, 1:100 dilution) and rabbit polyclonal anti-MMP9 (Abcam, 1:100 dilution). Diaminobenzidine was applied for color development, and hematoxylin was used for counterstaining.

### Hypoxia-induced zebrafish retinal angiogenesis

We selected 12- to 24-month-old zebrafish with EGFP-positive vessels in the retina for the experiments. Zebrafish in the control group were bred and reared normally, and zebrafish in the hypoxia-induced group were exposed to hypoxic water (10% air saturation) for 12 d. The 6G treatment groups were exposed to hypoxic water (10% air saturation for 12 d) with 15 or 30 μM of 6G. Many zebrafish were kept in the hypoxic water. After 12 d, the zebrafish were fixed at 4 °Covernight in 3.7% paraformaldehyde (solution volume/sample volume = 4:1). The zebrafish retina (attached to the lens) were removed with precision ophthalmic devices and then fixed on a glass slide. The detailed parameters and the dissecting and flatting methods applied to the adult zebrafish retinas have been previously described [25]. Finally, the samples were illuminated under low magnification at 488 nm using a Nikon confocal microscope (Nikon, Japan).

### Calculation of the fractal dimension

The fractal dimension of the retinal vasculature of adult zebrafish was calculated in MATLAB (MATLAB R2014b). Briefly, the image of retinal vasculature was loaded in MATLAB, and the green color was extracted. Then, the extracted binary data was further calculated using Box dimension in the Fractal module of MATLAB.

### Calculation of the coefficient of variation

The coefficient of variation is calculated by dividing the standard deviation by the mean. Use formula is expressed as: C.V. = σ/|μ|, σ = √∑(xi-u)^2/(n-1), u = (∑xi)/n.

### Microscale thermophoresis (MST)

The binding between 6G and VEGFR2 was analyzed using the MicroScale Thermophoresis Instrument Monolith NT.115 (NanoTemper Technologies, Munich, Germany). Briefly, the VEGFR2 protein was labeled with MST dye, and 6G was prepared using 16 geometric proportion serial dilutions (0.0046–150 μM). The labeled VEGFR2 and 6G working solutions were mixed and measured using an MST device. In the present study, the LED channel was set as “red” for the Cy5 dye, the temperature was set at 25 °C, and the MST was set at 40% power.

### Biacore assay

Surface plasmon resonance (SPR) experiments were performed using a Biacore 3000 instrument (GE Healthcare, Piscataway, NJ, USA). VEGFR2 was immobilized on CM5 sensor chips using the Biacore Amini Coupling Kit according to the manufacturer’s instructions. Different amounts of 6G were diluted and then injected into VEGFR2-immobilized CM5 sensor chips at concentrations of 6 μM, 12.5 μM, 25 μM and 50 μM. The corresponding concentration of 6G was set as control, and the background was subtracted. The surface of the control chip was prepared in the same manner and used for data correction. Data analysis was performed using BIA evaluation software.

### Scanning electron microscopy (SEM)

HUVECs grown on coverslips (KeyGen Biotech, Nanjing, China) were treated with solvent control, VEGFa or VEGFa/6G. After 12 h of incubation, the cells were fixed using ethyl alcohol, dried with a gradient concentration of tertiary butyl alcohol and finally coated with gold. Images of the cells were obtained using a scanning electron microscope (SEM, LEO1530VP, Germany).

### N-STORM microscopy of VEGFR2, VE-cad and β-catenin

Samples were prepared for single-molecule imaging to determine co-localization using super-resolution microscopy. HUVECs were seeded in 35-mm N-STORM super-resolution microscope dishes and grown to 60% confluence. The cells were cultured in RPMI-1640 medium (HyClone, USA) supplemented with 10% fetal bovine serum (HyClone) and antibiotics (50 units/mL penicillin and 50 μg/mL streptomycin). Next, the cells were cultured in the medium described above, medium containing 10 ng/mL VEGFa, or medium containing 10 ng/mL VEGFa and 15 μM 6G. The cells were incubated at 37 °C for 12 h in the presence of 5% CO_2_. The cells were fixed with 4% formalin for 30 min. Non-specific binding was blocked by incubation with 3% BSA for 30 min, and then the cells were incubated with rabbit polyclonal anti-VE-cad (Cell Signaling Technology (CST), USA), mouse polyclonal anti-β-catenin (CST) and goat polyclonal anti-VEGFR2 (Abcam, USA) antibodies in a humidified chamber at 4 °C overnight. Then, the cells were incubated with Alexa Fluor647-conjugated donkey anti-goat secondary antibody (1:200; Millipore) for 1 h at room temperature. Next, non-specific binding was blocked again by incubation with goat serum for 30 min. Then, the cells were incubated with Alexa 488-conjugated goat anti-rabbit secondary antibody and Alexa Fluor 568-conjugated goat anti-mouse secondary antibody for 1 h at room temperature. The N-STORM super-resolution microscope (Nikon, Japan) was used to assess the co-localization of VEGFR2, VE-cad and β-catenin at the nanoscale level.

### Proteomics analysis

HUVECs were seeded into a 60-mm dish. After reaching 70–80% confluence, the cells were treated with 6G (15 μM) for 12 h. Mass spectrometry was performed using a Q-Exactive Mass Spectrometer (Thermo Fisher Scientific). GO analyses for molecular functions, biological processes and cellular components were performed using ClueGo plug-in in CytoScape. Pathway analysis was performed using KEGG (Kyoto Encyclopedia of Genes and Genomes) databases.

### Molecular docking

An X-ray structure of the VEGF/VEGFR2 complex is available in the PDB database (PDBID: 3V2A). In the preparation steps, water molecules were removed, and the VEGFA/VEGFR2 crystal structure was treated as follows: hydrogen molecules were added, partial changes were assigned to relax amino residue side chains, and energy minimization was performed to relax the entire structure. The pocket of the VEGFA/VEGFR2 structure was selected to determine the active site of the docking grid box, and Glide XP (extra precision) was used for the 6G docking calculations.

### Patient-derived tumor xenograft (PDTX) models

Fresh tumor tissues were collected immediately after surgery from S. G. Hospital (Shandong, China), Tianjin Medical University General Hospital, and the Hospital of Shunyi District, Beijing. Written informed consent was obtained from each patient. The tumors were cut into 1–2 mm^3^ pieces in antibiotics-containing RPMI medium. Tumor fragments were implanted in subcutaneous pockets, which were made on each side of the lower back of BALB/c nude mice (4–6 weeks old). Samples with tumor sizes of 100–200 mm^3^ were called F0. Subsequently, the samples were divided for passaging in vivo to obtain F1 and then F2 xenograft tumors as described above [26, 27]. When the F2 tumor size reached 100–200 mm^3^, the mice were randomly divided into six groups (eight mice each) and treated with saline, cisplatin, sorafenib, sorafenib/cisplatin, 6G or 6G/cisplatin to serve as the xenograft tumor model.

### Statistical analysis

All data are expressed as the means ± standard deviations (SD). After testing for normality and equal variance across the groups, intergroup differences were assessed using Student’s t-tests, ANOVA, kaplan-meier survival analysis, GO and KEGG enrichment analysis. All experiments were repeated three times. *p < 0.05 or **p < 0.01 was considered statistically significant.

## Results

### Patients with cancer metastasis have higher MSE

**Fig. 1:**
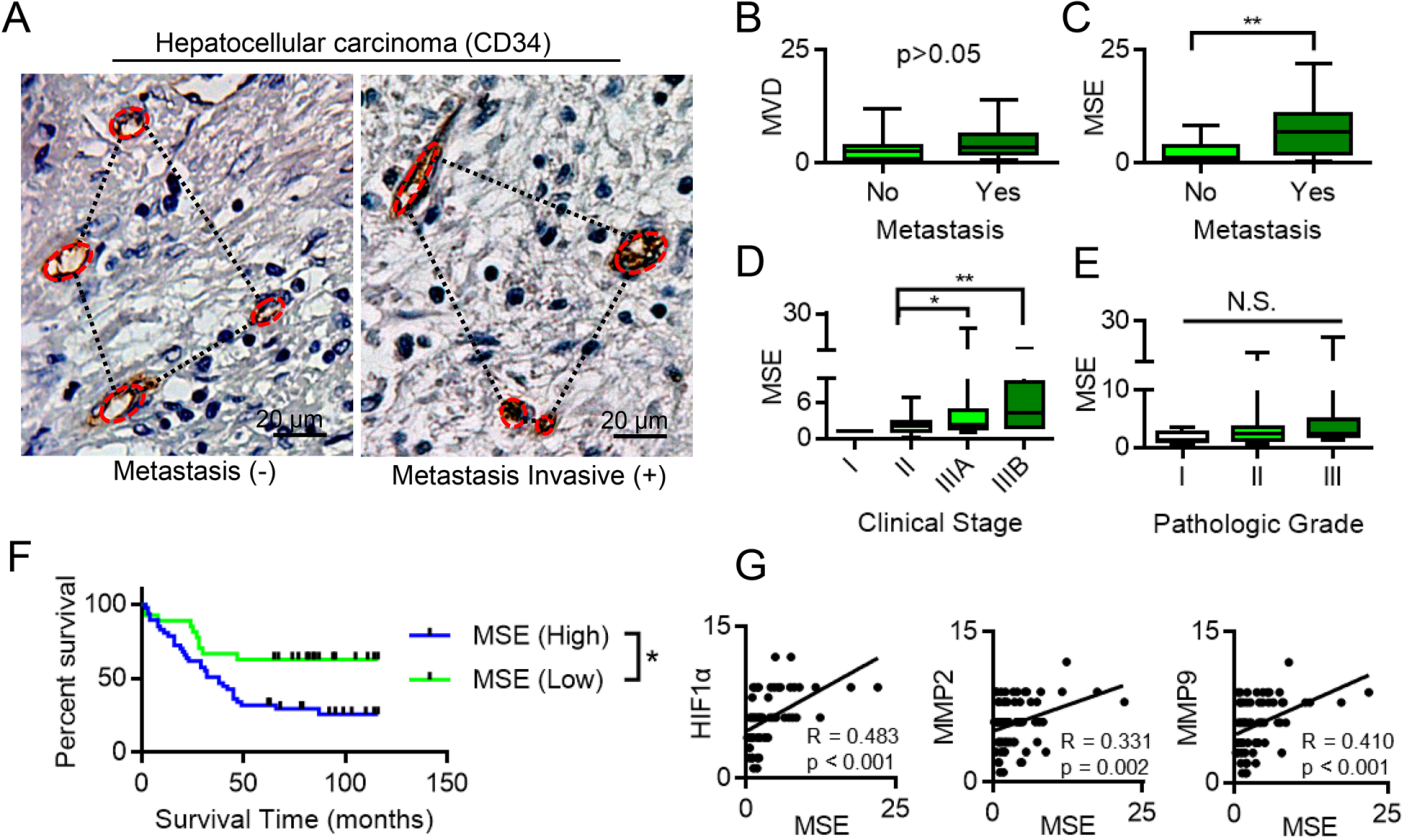
Microvascular structural entropy (MSE) might have potential as a biomarker of clinical outcomes. **a** Evaluation of MSE in liver cancer. **b** Relationship of metastasis and microvascular density (MVD) in 75 HCC tumor tissues, Student *t* test. **c** Pathological analysis the relationship of MSE and metastasis in 75 HCC tumor tissues. *, *P* < 0.05, Student *t* test. **d** Pathological analysis the relationship of MSE and clinical stage. *, P < 0.05; **, *P* < 0.01, one-way ANOVA. **e** Pathological analysis the relationship of MSE and pathologic grade. *, P < 0.05; **, *P* < 0.01, one-way ANOVA. **f** Kaplan–Meier plots of the overall survival rate of 75 patients with HCC with high MSE values and low MSE values group (P < 0.05). Results showed that patients with high MSE values have poor prognosis. **g** Correlation analysis of tumor tissues from 75 HCC cases between MSE and HIF1α, MMP2 and MMP9. MSE was positively correlated with HIF1α, MMP2 and MMP9 levels. Intergroup differences were assessed using Student’s t-tests. For all data, N.S., not significant, *P < 0.05, **P < 0.01

MSE, a measure of the degree of disorder, may be used as a tumor biomarker in hepatocellular carcinoma (HCC). Patients with liver metastasis have higher MSE (Fig. 1a). In histopathological analyses of liver cancer, MSE was positive correlation with metastasis (Fig. 1b and c). A greater tortuosity and branching in tumor microvessels can increase the likelihood of tumor invasion and metastasis. MSE is a more sensitive marker than is MVD. Furthermore, MSE was associated with clinical stage (TNM staging system was adopted), but not correlation with pathologic grade (Three-stage method) (Fig. 1d and e) [28]. High MSE was correlated with low survival time in HCC patients (Fig. 1f), indicating that more irregular and tortuous microvessels with high MSE values can lead to a poor prognosis. In tumor cells, MSE is positively correlated with HIF1α, MMP2 and MMP9 expression and thus can reflect the status of the tumor microenvironment (Fig. 1g). Therefore, MSE may be reflective of tumor behavior and could be used as an independent prognostic indicator in the clinic.

### 6G decreased MSE to improve the tumor microenvironment and suppress tumor progression

**Fig. 2:**
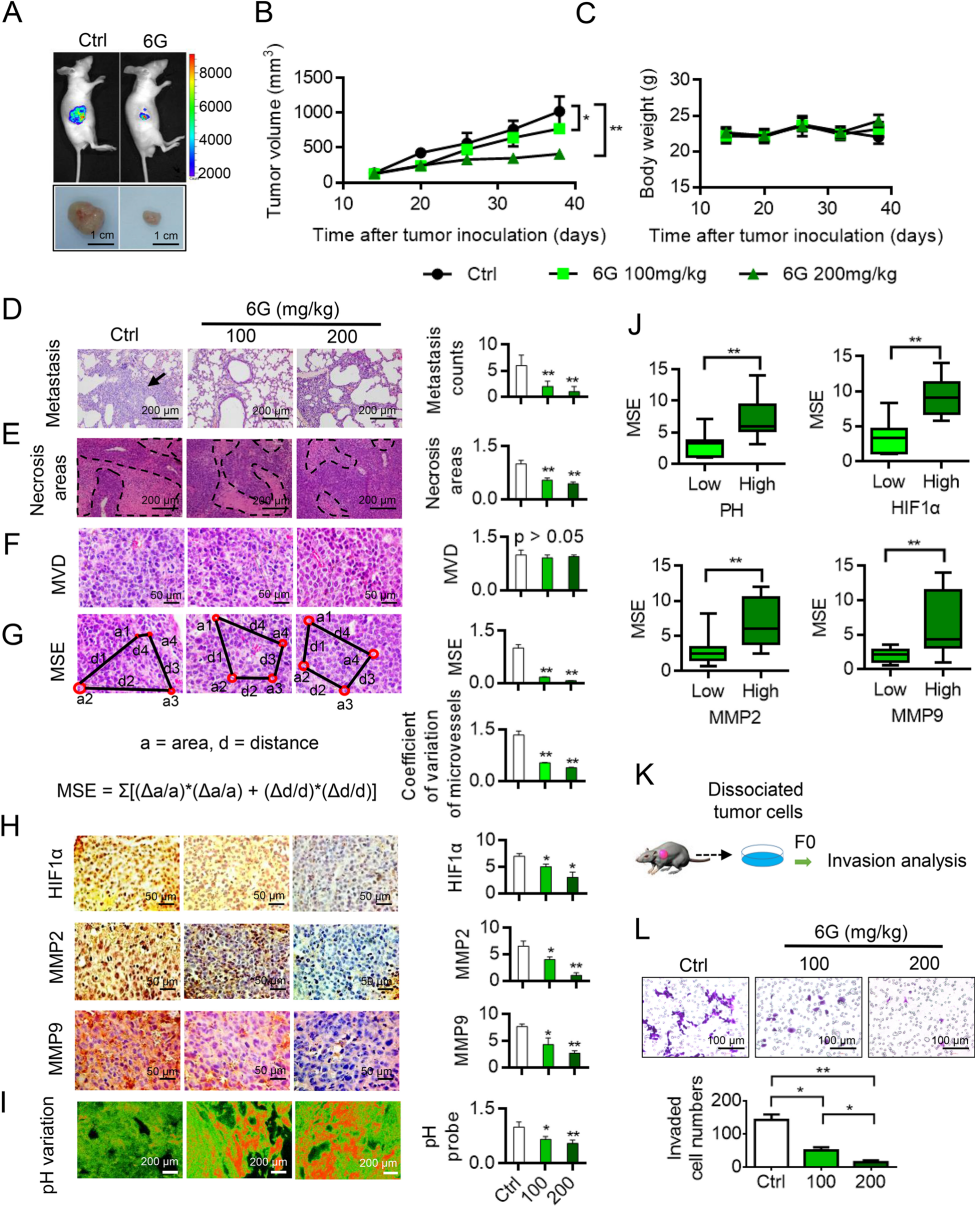
6G suppresses the growth and metastasis, improves the tumor microenvironment and decreased MSE in vivo. **a** and (**b**) Representative tumors and in vivo bioluminescent images and tumor volume are shown. Each point represents the mean ± SD for different animal measurements (*n* = 10). *, *P* < 0.05; **, *P* < 0.01, one-way ANOVA. 6G inhibited TCs proliferation with dose-dependent manner. **c** Mice weight of each groups. **d** Representative lung metastasis specimens were sectioned and stained with **h**&**e** in 6G-treated group and control group. *, P < 0.05; **, P < 0.01, one-way ANOVA. (E) Representative images of the necrotic areas. 6G reduced the necrotic area. *, P < 0.05; **, P < 0.01, one-way ANOVA. **f** MVD was not changed between 6G-treated and control groups. *, P < 0.05; **, P < 0.01, one-way ANOVA. **g** Representative images, MSE and coefficients of variation of microvessels in 6G-treated and control group. *, *P* < 0.05; **, *P* < 0.01, one-way ANOVA. (H) 6G-treated sections demonstrated weaker HIF1α, MMP2 and MMP9 expression than did untreated sections. *, P < 0.05; **, P < 0.01, one-way ANOVA. **i** pH changes in the 6G-treated and untreated tumors. 6G increased the pH of the local tumor tissue and improved the microenvironment surrounding TCs. *, P < 0.05; **, P < 0.01, one-way ANOVA. **j** MSE was correlated with pH, HIF1α, MMP2 and MMP9 in tumor. *, *P* < 0.05, **, *P* < 0.01, Student *t* test (**k**) and (**l**) Invasion analysis of 6G effect on primary tumor cells. *, P < 0.05; **, P < 0.01, one-way ANOVA. 6G inhibited tumor malignancy. Results were obtained from three independent experiments, each performed in triplicate, and the error bars represent SD. Data are represented as the means ± SEM

Ginger has been used for thousands of years in China as an “interior-warming drug”. Ginger has the ability to alleviate gastrointestinal paralysis and spasms and improve dysmenorrhea and cold stress. As the major bioactive component in ginger, 6G has been reported to influence microcirculation. Therefore, we analyzed the anti-tumor effects of 6G. Treatment with 6G promoted a reduction in tumor volume of mice in a dose-dependent manner. There was no significant change in animal weight in 6G treated group (Figs. 2a and c). Compared to the control group, 6G-treated group exhibited reduced metastatic burden (Fig. 2d) and reduced the necrotic areas (Fig. 2e). 6G did not affect microvessel density (MVD) (Figs. 2f). Compared with the control group, 6G decreased MSE and the coefficient of variation among microvessels (Fig. 2g). Immunohistochemical (IHC) staining demonstrated a reduction in the expression of HIF1α, MMP2 and MMP9 in the 6G-treated group than in the untreated group (Fig. 2h). Using a pH probe that changes color from green to yellow with pH values of 6.2 and 7.5, respectively. 6G was found to increase the pH of the tumor microenvironment (Fig. 2i). Furthermore, MSE was associated with pH and HIF1α, MMP2 and MMP9 levels (Fig. 2j). Cancer cells were isolated from xenografts and cultured in vitro for the evaluations of their migratory ability. Results showed that 6G inhibited the migration of tumor cells (Fig. 2k and l).

### 6G promotes retinopathy vessel normalization in hypoxia-induced zebrafish model

**Fig. 3:**
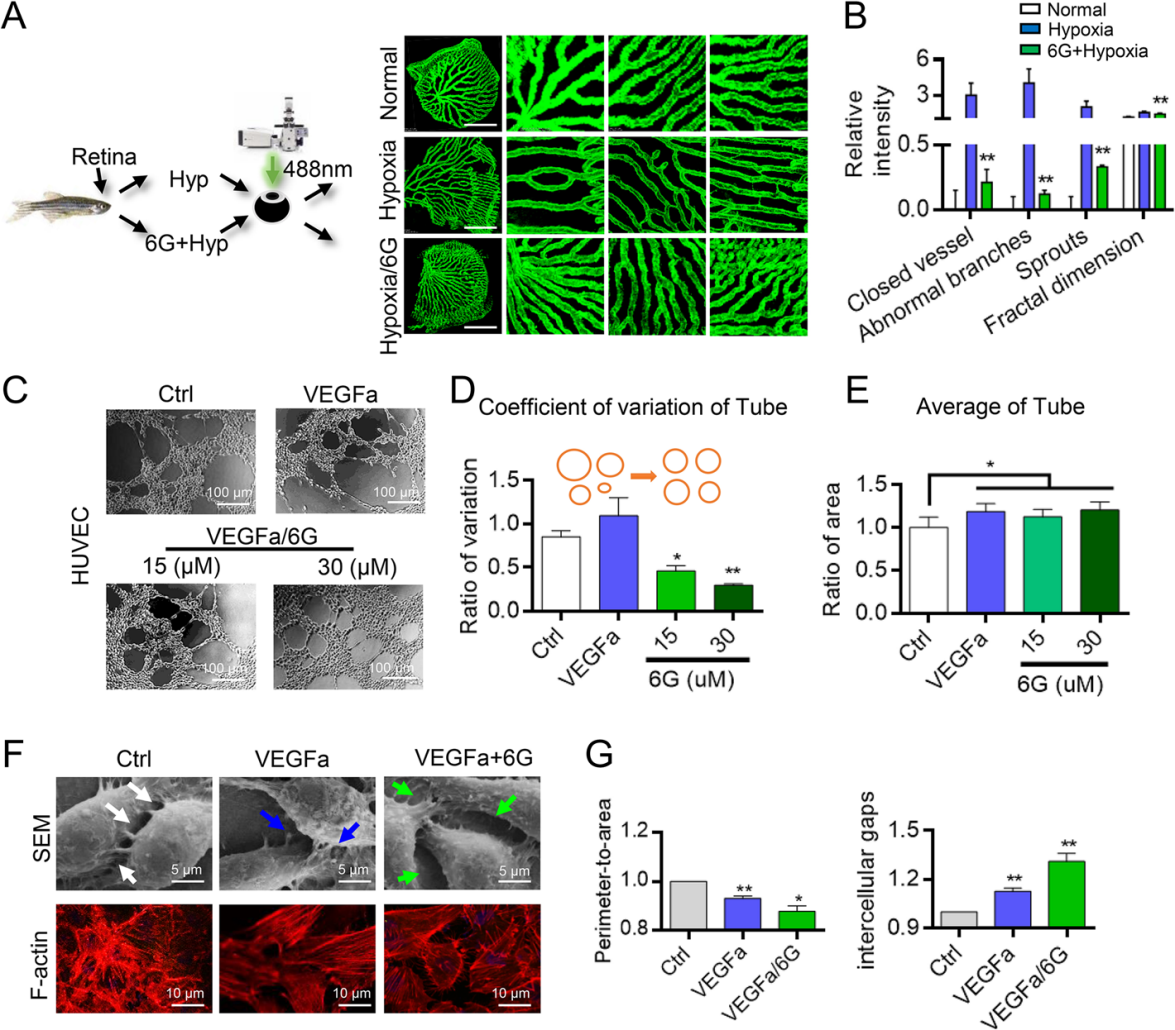
Effect of 6G on vascular normalization in zebrafish model and vascular remodeling in HUVECs. **a** Representative zebrafish retinal angiogenesis in normal, hypoxia and hypoxia/6G group. **, *P* < 0.01, one-way ANOVA. **b** Closed vessel, abnormal branches, sprouts and fractal dimension values in normal, hypoxia and hypoxia/6G group of zebrafish retinal vessels. *, P < 0.05; **, P < 0.01, one-way ANOVA. 6G exerted a protective effect against hypoxic damage. **c** Effect of 6G on tube formation in HUVECs. *, P < 0.05; one-way ANOVA. **d** Coefficient of variation of tube. 6G decreased the coefficient of variation among tubes. **e** Average area of tube. 6G increased tube area. **f** SEM and fluorescence staining showing the effect of 6G on HUVECs. **g** 6G promotes endothelial cytoskeleton remodeling and regulates pseudopod connections of HUVECs. *, *P* < 0.05; **, *P* < 0.01, one-way ANOVA. Results were obtained from three independent experiments, each performed in triplicate, and the error bars represent SD. Data are represented as the means ± SEM

To further identify the relationship between the 6G and microenvironment, we employed the adult zebrafish hypoxia-induced retinopathy model. Compared with the control group, the capillary bed of the retina exhibited minor alterations and marked angiogenesis in the hypoxia-induced group (Fig. 3a). The angiogenic responses were separately quantified in each region, and the vascular fractal dimension was calculated using an algorithm for averaging grayscale values. The fractal dimension reflects the chaos of a system and is a 3D representation of the 2D measure of “MSE”, the calculation process of fractal dimension showed at Additional file 1: Figure S1. Addition of 6G for 12 days in the water reversed vascular injuries induced by hypoxia and reduced the fractal dimension in the zebrafish (Fig. 3b).

Next, tube formation assays using HUVECs were performed to examine the changes of vascular fractal dimension. Compared with the control and VEGFa group, the tubes in the 6G-treated group were uniformly distributed (Fig. 3c). Moreover, the coefficient of variation among the tubes decreased, but the average tube area was not altered in response to 6G (Fig. 3d and e). To further determine the mechanism by which 6G alters the microenvironment, the scanning electron microscopy (SEM) and cytoskeletal labeling assays were performed. The results showed that the 6G regulates remodeling of endothelial cytoskeleton (Fig. 3f). 6G enhance the remodeling of cytoskeleton and reduced the pseudopodium, whereas it increased intercellular pores and promoted F-actin re-arrangement (Fig. 3g).

### 6G enhances the activating effects of VEGFa/VEGFR2

**Fig. 4:**
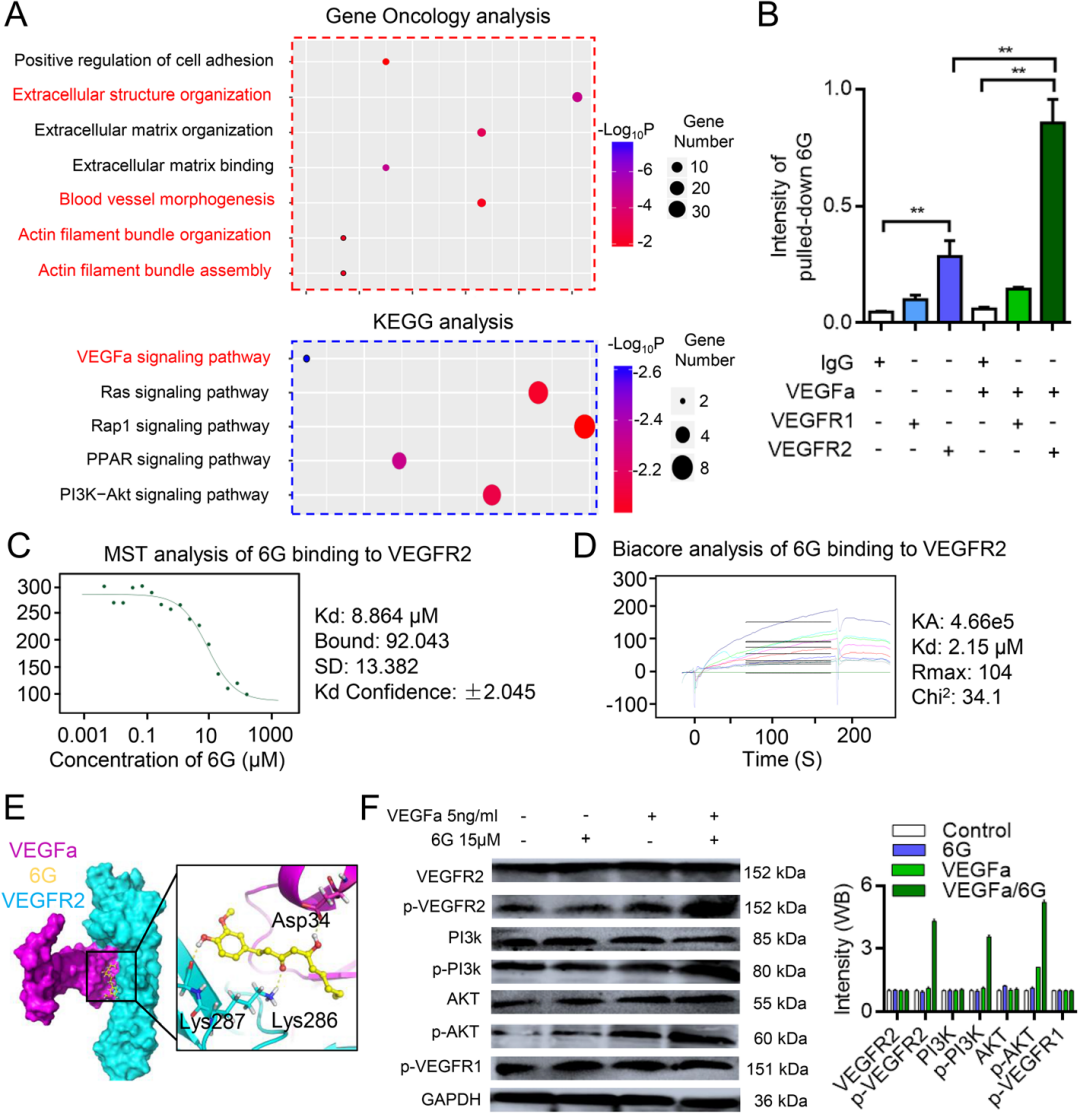
6G influences the VEGF/VEGFR2 signaling pathway and targeting of VEGFR2. **a** Gene oncology and KEGG enrichment analysis of 6G-treated and control HUVECs. Enrichment analysis of differentially expressed proteins revealed associations with the extracellular structure organization, blood vessel morphogenesis, actin filament bundle organization and assembly. Pathway analysis of 6G-affected proteins showed that 6G targeted the VEGFa/VEGFR2 pathway. **b** Positive pull-down assay confirming the binding of 6G with VEGFR2 but not with VEGFR1. **, P < 0.01, one-way ANOVA. **c** Microscale thermophoresis (MST) assay showing the binding of 6G to VEGFR2. **d** Surface plasma resonance assay confirming the interaction between 6G and VEGFR2. 6G targeted VEGFR2 with favorable binding results. **e** Molecular docking of 6G binding into the active site of VEGF/VEGFR2 complex. 6G forms one key hydrogen bond with VEGFa and two hydrogen bonds with VEGFR2. **f** Effect of 6G on p-VEGFR2, p-PI3k and p-AKT in HUVECs. Results were obtained from three independent experiments, each performed in triplicate, and the error bars represent SD (^*^P < 0.05, ^**^P < 0.01)

Proteomics analysis was performed in 6G-treated and control HUVECs. The Gene Ontology (GO) analysis showed that 6G enhances extracellular structure organization, blood vessel morphogenesis and actin filament bundle organization. KEGG enrichment analysis revealed that the differentially expressed proteins affected by 6G were enriched in the VEGFa signaling pathway (Fig. 4a and Additional file 1: Figure S2). Pathway analysis indicated that 6G targeted VEGFR2 and influenced the expression and functions of VEGFR2 downstream proteins. Direct pull-down assays followed by the detection of 6G using its auto-fluorescence intensity were performed to confirm the binding of 6G to VEGFR2 (Additional file 1: Figure S3). The intensity of 6G was not different between cells that were treated with or without VEGFa. However, in response to pretreatment with VEGFa, the intensity of 6G fluorescence was higher in the VEGFR2-pull-down group than that in the VEGFR1-pull-down and control groups (Figs. 4b). The binding of 6G to VEGFR2 was further confirmed using microscale thermophoresis (MST) and the Biacore assay. The curve for 6G vs VEGFR2 exhibited a good fit, with a dissociation constant (K_D_) of 8.86 μM in MST experiment and 2.15 μM in Biacore assay (Figs. 4c and d). Molecular docking (MD) were performed to assess the binding mode of 6G with the VEGFa/VEGFR2 complex. The docking score of 6G on the VEGFa/VEGFR2 complex was − 8.064, which indicated suitable interactions (Fig. 4e). The binding model revealed that 6G stretched out and linked VEGFa and VEGFR2 together in a rope-like manner. 6G bound to the active site of VEGFa/VEGFR2 in the form of a “chain” and formed one key hydrogen bond with VEGFa (Asp34) and two hydrogen bonds with VEGFR2 (Lys286 and Lys287).

To identify whether 6G acts as an agonist or antagonist to VEGFR2, western blotting was performed to analyze the expression of VEGFR2 and its downstream proteins. 6G did not alter the expression of VEGFR1 and p-VEGFR1. However, the phosphorylation of VEGFR2 was increased in the presence of VEGFa. Additionally, compared to the control group, although the proteins downstream of VEGFR2 (AKT and PI3k) were not affected, the levels of p-AKT and p-PI3k were increased in the 6G-treated group (Fig. 4f). Therefore, 6G acts as an agonist to VEGFR2. Although 6G can regulate VEGFa/VEGFR2 signaling pathway, the pathway opening cannot affect cytoskeletal remodeling. Therefore, we speculate that 6G plays a role in regulating cytoskeleton, possibly through a non-pathway-dependent mechanism.

### P-VEGFR2/VE-cad/β-catenin/actin complex regulates vascular remodeling and improves the tumor microenvironment

**Fig. 5:**
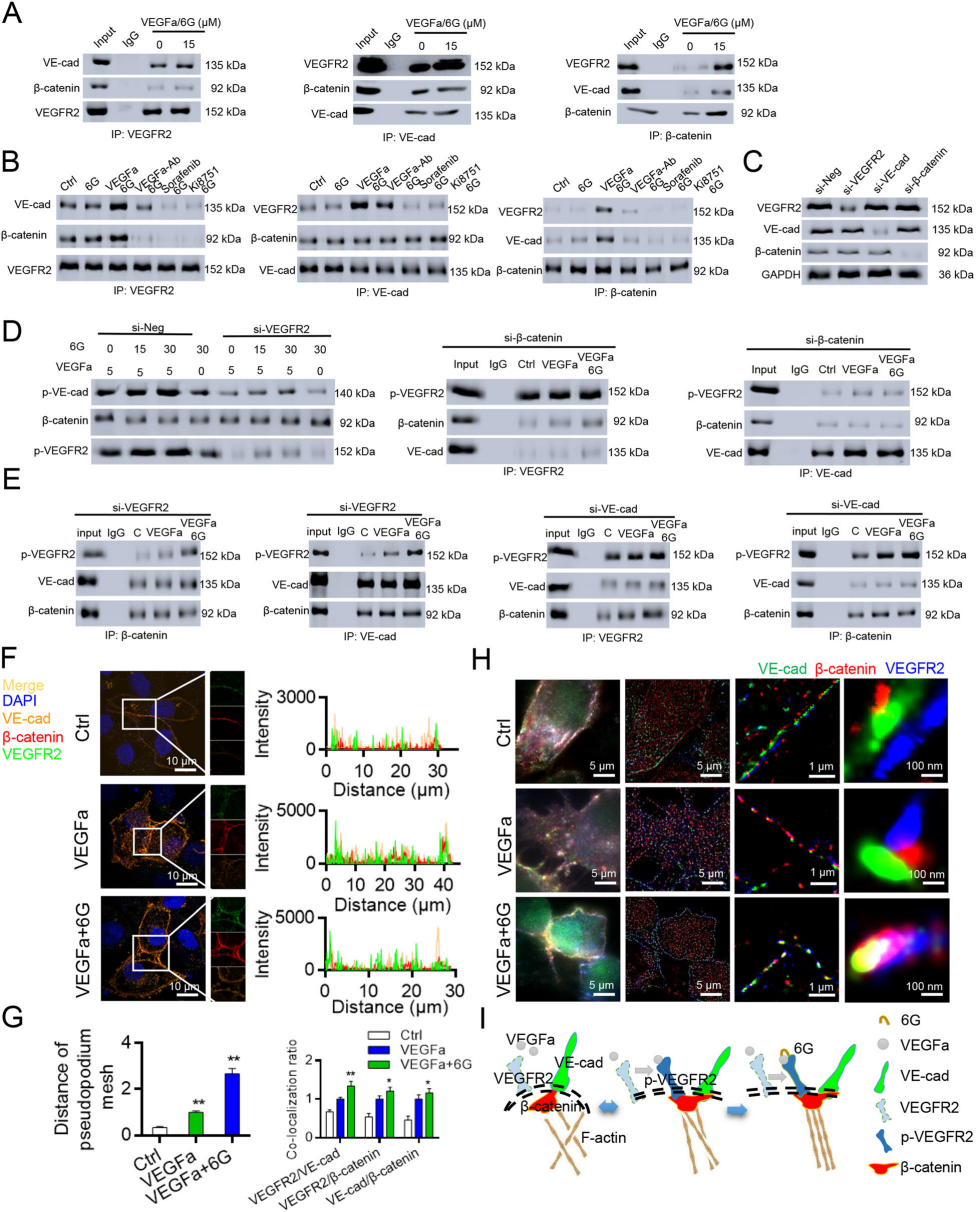
6G increases the interaction between VEGFR2 and VE-cad/β-catenin and enhances complex-regulated F-actin remodeling. **a** VEGFR2/VE-cad complex strongly interacted with β-catenin in response to VEGFa, and 6G increased this interaction. **b** 6G increased the phosphorylation of VEGFR2 and increased its interaction with VE-cad. **c** Knock down VEGFR2, VE-cadherin and β-catenin in HUVECs. There was no correlation between the expression of VEGFR2, VE-cad and β-catenin. **e** VEGFR2, VE-cad and β-catenin physically interact with one another, and β-catenin acts as a “bridge” to connect activated VEGFR2 and VE-cad. **f** Confocal image of VE-cad, VEGFR2 and β-catenin. Results demonstrated that there were significant co-localization of VE-cad, VEGFR2 and β-catenin in the cell membrane. **g** Distance of pseudopodium and co-localization ratio of cells in control, VEGFa and VEGFa/6G group. *, P < 0.05; **, P < 0.01, one-way ANOVA. **h** Single-molecule imaging of VEGFR2, VE-cad and β-catenin. VEGFR2/VE-cad/β-catenin complex was formed at the cell membrane, and this was enhanced by 6G. **i** Schematic showed the interaction of p-VEGFR2 with VE-cad and β-catenin induced alterations in F-actin organization to regulate cellular extensions in HUVECs. Results were obtained from three independent experiments, each performed in triplicate, and the error bars represent SD (^*^P < 0.05, ^**^P < 0.01)

In a study on podocytes, the mechanism of regulation of vascular remodeling was dependent on the interaction between VEGFR2 and nephrin [29]. Nephrin is an epithelial cell adhesion molecule. Thus, we hypothesized that the remodeling of HUVECs was regulated in a similar fashion to that of podocytes. VEGFR2 has been reported to interact with VE-cad, an endothelial cell adhesion molecule, but the function of the resulting protein complex remains unknown [30, 31]. Using immunoprecipitation (IP) experiments, we found that 6G increases the interaction between VEGFR2 and VE-cad. Interestingly, the VEGFR2/VE-cad complex strongly interacts with β-catenin in response to VEGFa, and treatment with 6G further enhances this interaction (Fig. 5a).

IP experiments were also performed to evaluate interaction of VEGFR2 with β-catenin and VE-cad in HUVECs. Reciprocal co-IP experiments confirmed that VEGFR2 interacted with β-catenin and VE-cad to form a complex in cultured HUVECs. To evaluate the importance of VEGFa in the formation of the VEGFR2/VE-cad protein complex, we performed a series of co-IP experiments in the presence of anti-VEGFA antibodies (VEGFa-Ab), sorafenib and Ki8751. The phosphorylation of VEGFR2 and formation of the VEGFR2/VE-cad complex was inhibited in response to VEGFa-Ab, sorafenib and Ki8751 (Fig. 5b).

To determine whether the interactions of proteins in the complex are regulated via specific pathway-related protein expression, we used siRNAs to knockdown VEGFR2, VE-cad, and β-catenin. Indeed, the siRNAs were effective in knocking down the respective proteins. However, the expression levels of the three proteins were not affected by one another (Fig. 5c). The interactions among VE-cad, VEGFR2 and β-catenin were not regulated at the level of protein expression but rather via physical contacts. To assess the mode of interaction among the proteins, we used siRNAs to knockdown VEGFR2, VE-cad, and β-catenin. Knockdown of VEGFR2 resulted in a decrease in the phosphorylation of VE-cad. Therefore, VEGFR2 is likely involved in the phosphorylation of VE-cad (Figs. 5d and e).

We demonstrated that β-catenin was working as a “bridge” to connect activated VEGFR2 and VE-cad. 6G did not affect the interactions between VE-cad and β-catenin.VE-cad, VEGFR2, and β-catenin were co-localized to the cell membrane (Fig. 5f). In response to VEGFa, the co-localization coefficient increased from 0.805 to 0.861, and 6G further increased this value to 0.904 (Fig. 5g). We used single-molecule imaging with “N-STORM” to analyze the conformation of the three interacting proteins at 2-nm resolution (Fig. 5h). Results showed that 6G promoted the interaction of VEGFR2, VE-cad and β-catenin in HUVECs. Figure 5i demonstrates the interaction between VEGFR2 and VE-cad, activation of VEGFR2 phosphorylation by VEGFa and interaction between VE-cad and β-catenin, and subsequent regulation of cell surface tension by the protein complex. Additionally, the F-actin arrangement changed from anisotropic to isotropic. Therefore, VEGFa activates VEGFR2 and forms the VEGFa/VEGFR2/VE-cad/β-catenin/actin complex, which regulates cell cytoskeletal networks, and 6G enhances this process.

### 6G improve tumor microenvironment and enhance antitumor activity of cisplatin

**Fig. 6:**
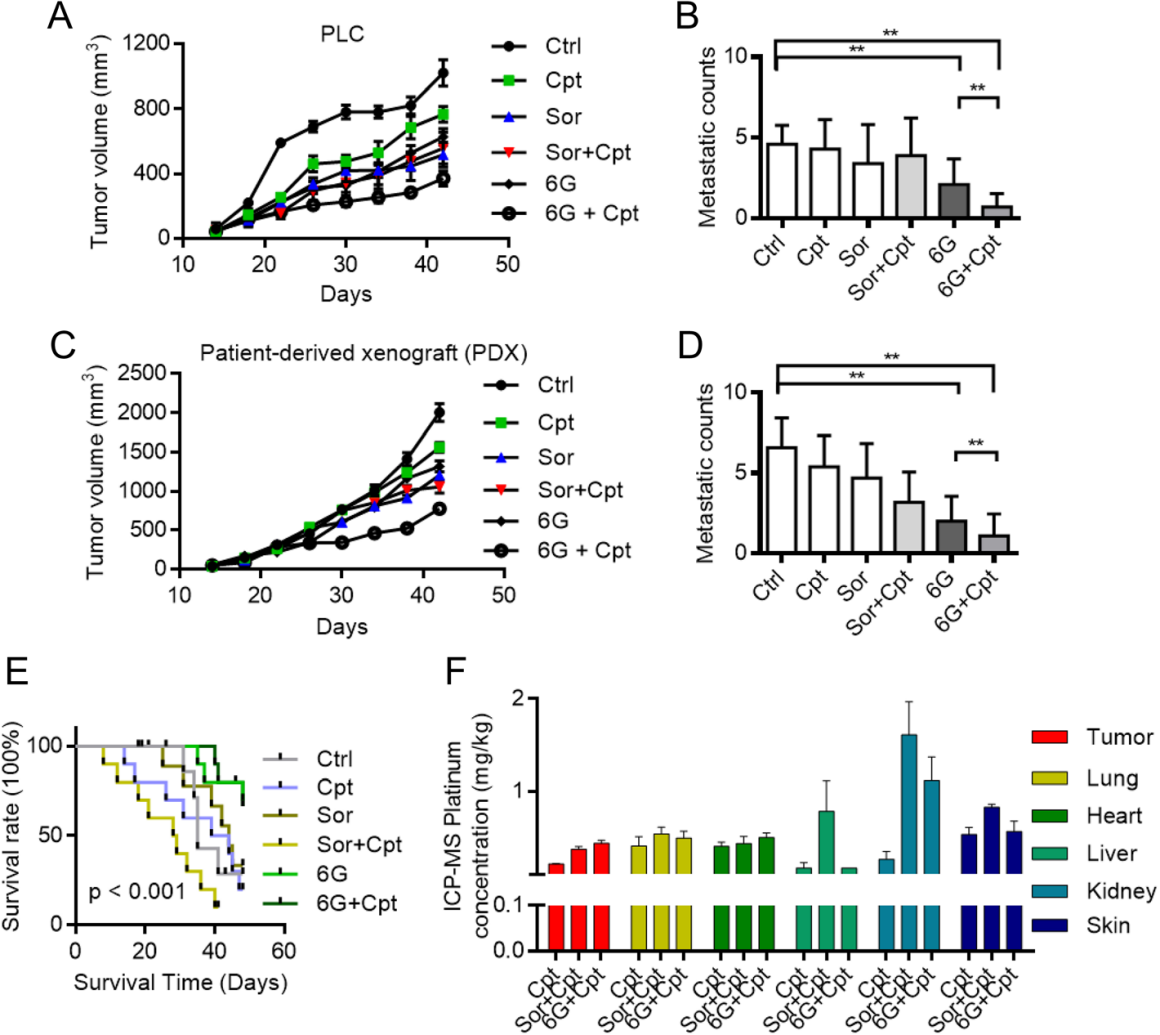
Effect of 6G in CDX and PDX models. **a** and (**b**) 6G enhance the antitumor effect of cisplatin and inhibited the growth and metastasis in PLC/PRF/5 xenografts model (*n* = 8). **, P < 0.01, one-way ANOVA. **c** and (**d**) 6G enhanced the anti-tumor effect of cisplatin and reduce metastasis in Patient-derived xenograft (PDX) model (*n* = 8). **, P < 0.01, one-way ANOVA. **e** 6G combination of cisplatin increased the survival time of mice, while the anti-angiogenesis drugs sorafenib combination of cisplatin decreased survival time. **f** Inductively coupled plasma mass spectrometry (ICP-MS) analysis the distribution of metal platinum in various organs of mice. Results showed that 6G increased the content of metal platinum in tumor. While, sorafenib reduces the distribution of metal platinum in tumors, but increases the distribution of metal platinum in the liver, kidney and skin. Results represent the means of three experiments, and all values represent the means ± SEM. ^*^P < 0.05, ^**^P < 0.01

6G exhibited obvious anti-tumor effects in vivo. Results of PLC/PRF/5 cell-derived xenograft (CDX) showed that compared with the untreated group, the 6G and cisplatin co-treatment group demonstrated inhibited tumor growth (Fig. 6a). Furthermore, metastasis was decreased in the 6G/cisplatin group (Figs. 6b). The same results was also observed in the patient-derived xenograft (PDX) models (Fig. 6c and d).

Therefore, 6G plays an anti-tumor role by regulating the tumor microenvironment, and increasing the therapeutic efficacy of cisplatin and suppressing metastasis both in vitro and in vivo. We performed survival experiments using combination treatment with the VEGFR2 inhibitor sorafenib and the chemotherapeutic agent cisplatin. The combination of sorafenib and cisplatin shortened the survival time (Fig. 6e).

Inductively coupled plasma-MS (ICP-MS) showed that 6G promote cisplatin delivery into tumor tissues and thus increased tumor sensitivity to cisplatin while reducing systemic toxicity. However, sorafenib inhibited angiogenesis and vascular permeability, thus decreasing the delivery of cisplatin into tumors. This resulted in the distribution of cisplatin to the organs of the whole body, resulting in systematic injury (Fig. 6f). Reactive oxygen species (ROS) and the dead/live cell ratio were increased in the 6G and cisplatin co-treatment group of PLC/PRF/5 cell lines (Additional file 1: Figure S4).

## Discussion

The growth and metastasis of malignant tumors depends on an adequate blood supply, which can be achieved by the recruitment of vessels to establish microcirculation in tumors. Some factors, such as hypoxia, IFP and pH, can affect tumor microcirculation [32]. A hypoxic microenvironment and excess IFP within the tumor can affect microcirculation and promote an aggressive behavior along with the acquisition of TC stemness. Several critical signaling pathways have been identified to have important roles in tumor angiogenesis, including the PI3k/Akt/mTOR, NF-휅B, Notch, Wnt/훽-catenin and Hedgehog signaling pathways.

Previous data provide evidence that MVD does not fully mirror the interaction between blood supply and TC proliferation [24]. To evaluate tumor vasculature normalization, an independent parameter, MSE, was calculated using histological features to measure microvascular heterogeneity. Histopathological evaluation of liver cancer demonstrated that MSE was associated with clinical stage and clinical prognosis. Previous studies have focused more on the number and area of vessels, but not the MSE. Tumor metastasis is related to MSE but not to MVD. The MSE may be an important parameter of vascular normalization.

6G is a polyphenolic compound derived from ginger that demonstrates anti-tumor effects in multiple tumors [33]. In the present study, 6G exerted anti-tumor effects and decreased MSE by promoting vascular normalization. VEGFR1 regulates the growth of ECs and the extension and repair of vessels, whereas VEGFR2 regulates the differentiation of ECs and the remodeling of vessels [34, 35]. 6G increased the binding of VEGFa with VEGFR2 and maintained VEGFR2 phosphorylation. However, it did not affect the binding of VEGFa to VEGFR1. While, the effect of 6G on the remodeling of ECs was not dependent on the PI3K/Akt pathway, but depend on p-VEGFR2/VE-cad/β-catenin/actin protein complex. 6G binds to the VEGFa/VEGFR2 complex and induces conformational changes in VEGFR2, resulting in its dimerization and autophosphorylation to p-VEGFR2. Thus, the interaction among the members of the p-VEGFR2/VE-cad/β-catenin/actin protein complex was modulated by tyrosine phosphorylation and the subsequent recruitment of β-catenin by p-VEGFR2, leading to actin polymerization and stress fiber formation, which eventually regulated the migration of ECs. Indeed, 6G induced the p-VEGFR2/VE-cad/β-catenin/actin complex formation and the actin cytoskeletal remodeling to increase the supply of oxygen and nutrients to TCs by normalizing the tumor vasculature, resulting in alleviated microenvironmental stress and prevented tumor progression. 6G also shown to enhance the delivery of cisplatin into the tumor core via increased permeability and normalized tumor vasculature [36]. Both CDX and PDX models showed that compared with the untreated group, the 6G and cisplatin co-treatment group displayed inhibited tumor growth. 6G increased the distribution of oxygen and chemotherapeutic drugs in tumors, leading to profound anti-tumor effects by increasing ROS to directly or indirectly kill the TCs. In addition, published data have demonstrated the benefit of ginger over placebo in Functional Living Index Emesis nausea score (day 6-day 1) for female patients (*P* = 0.048) and head and neck cancer (HNC) patients (*P* = 0.038). The results showed that the daily use of ginger is safe in patients with high dose cisplatin therapy. Similarly, ginger shows good results in specific nausea risk subgroups, such as in female patients and HNC patients [37].

The “feeding” and “starvation” of TCs are the two sides of a coin that can influence the choice of tumor therapy. Because of disrupted structure and function of vessels and high IFP, tumors are poorly perfused with blood, leading to extreme hypoxia and drug resistance and increased microenvironmental stress. These alterations in turn induce the progression, invasion and metastasis of TCs. Anti-angiogenesis drugs are designed to inhibit blood supply to tumors. However, many of these agents often promote tumor progression despite decreasing tumor volume, thus leading to increased metastasis [19, 38]. Many investigators have shown that the anti-angiogenesis drugs, such as sorafenib can cause tumor recurrence and metastasis. This phenomenon has become an important bottleneck in anti-vascular treatment. Interestingly, we also reported for the first time that anti-angiogenesis drugs decrease MVD but increase MSE and thus may be associated with tumor metastasis. Therefore, MSE might have potential as a biomarker to assess the proliferation and metastasis of TCs. Appropriate “feeding” of TC scan reduce stress on the tumor microenvironment. Although this view contradicts the idea of anti-vascular therapy and has not yet been fully recognized, some studies on the tumor microenvironment, immunity, and metabolism have shown that “feeding” TCs may not be wrong. In this study, 6G increased the remodeling of tumor vasculature and prevented distant metastasis. Thus, the strategy of increasing the remodeling of ECs in tumors to enhance the delivery of chemotherapeutic drugs and oxygen into the tumor core and to generate a favorable tumor microenvironment may be a promising therapeutic approach in the clinic. Normalization of the tumor vasculature can affect HIF1α/VEGFa feedback and reduce TC recruitment or vasculogenic mimicry (VM) formation, leading to better clinical responses in cancer patients [39, 40].

## Conclusions

In summary, we reported that MSE may be used a biomarker associated with tumor metastasis, prognosis, and chemotherapy response. 6G exhibits a therapeutic effect by reduce MSE and regulating the p-VEGFR2/VE-cad/β-catenin/actin complex to affect pseudopodial structures in ECs. 6G improves tumor microenvironment, reduces microenvironmental stress and promotes the normalization of tumor vessels, thereby inhibiting the progression, invasion and metastasis of tumors. In the present study, we showed that the normalization of tumor vessels improved the microenvironment to amplify the effect of chemotherapeutic drugs. Our results will contribute to improve anti-angiogenesis therapy by providing a new direction for the combinatorial treatment of cancer and increasing the current understanding of the pharmacological mechanisms of ginger.

## Additional file

Additional file 1: **Figure S1.** The calculation process of fractal dimension. **Figure S2.** Pathway analysis revealed that 6G influenced VEGFa/VEGFR2 pathways. **Figure S3.** Positive pull-down assay of 6G with VEGFR1 and VEGFR2. **Figure S4.** Flow cytometry analysis of 6G treated and untreated cells. Results showed that ROS levels and dead cells were significantly increased in the 6-gingerol and cisplatin co-treatment group. (DOCX 609 kb)

## Data Availability

The datasets used and/or analyzed in the current study are available from the corresponding author on reasonable request.

## Abbreviations

6G: 6-Gingerol
CDX: cell-derived xenograft
EC: endothelial cell
EMT: epithelial-mesenchymal transition
HCC: hepatocellular carcinoma
ICP-MS: inductively coupled plasma-MS
IHC: immunohistochemistry
MSE: microvascular structural entropy;
MST: microscale thermophoresis
MVD: microvessel density
PDX: patient-derived xenograft
SEM: Scanning electron microscopy
STORM: stochastic optical reconstruction microscopy
TC: tumor cell
