# Supplementary material for: 6-Gingerol stabilized the p-VEGFR2/VE-cadherin/β-catenin/actin complex promotes microvessel normalization and suppresses tumor progression
Source: J Exp Clin Cancer Res. 2019 Jul 2;38:285. doi: 10.1186/s13046-019-1291-z (PMC6604152; doi:10.1186/s13046-019-1291-z)
Supplement: Supplementary file 1 — Figure S1. The calculation process of fractal dimension. Figure S2. Pathway analysis revealed that 6G influenced VEGFa/VEGFR2 pathways. Figure S3. Positive pull-down assay of 6G with VEGFR1 and VEGFR2. Figure S4. Flow cytometry analysis of 6G treated and untreated cells. Results showed that ROS levels and dead cells were significantly increased in the 6-gingerol and cisplatin co-treatment group. (DOCX 609 kb) [file 13046_2019_1291_MOESM1_ESM.docx]

**Supplementary data**

**6-Gingerol stabilized the p-VEGFR2/VE-cadherin/β-catenin/actin complex promotes microvessel normalization and suppresses tumor progression**

Weilong Zhong^2#^, Wendong Yang^1,3#^, Yuan Qin^1,3^, Wenguang Gu^1^, Yinyin Xue^1,3^, Yuanhao Tang^1,3^, Hengwei Xu^1,3^, Hongzhi Wang^1,3^, Chao Zhang^1,3^, Changhua Wang^1^, Bo Sun^3^, Yanrong Liu^3^, Huijuan Liu^1^, Honggang Zhou^1,3^, Shuang Chen^3*^, Tao Sun^1,3*^, Cheng Yang^1,3*^

^1^State Key Laboratory of Medicinal Chemical Biology and College of Pharmacy, Nankai University, Tianjin, 300350, China.

^2^Department of Gastroenterology and Hepatology, Tianjin Medical University General Hospital, Tianjin Institute of Digestive Disease, Tianjin 300052, China.

^3^Tianjin Key Laboratory of Molecular Drug Research, Tianjin International Joint Academy of Biomedicine, Tianjin, 300000, China

# These authors contributed equally to this work.

Corresponding Author: Telephone: +86-022-85358658; Shuang Chen: shuang7332@163.com; Tao Sun: sunrockmia@hotmail.com; Cheng Yang: cheng.yang@nankai.edu.cn.

SUPPLEMENTARY FIGURES


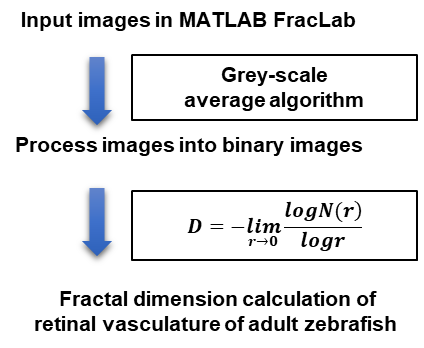


**Figure S1.** The calculation process of fractal dimension.


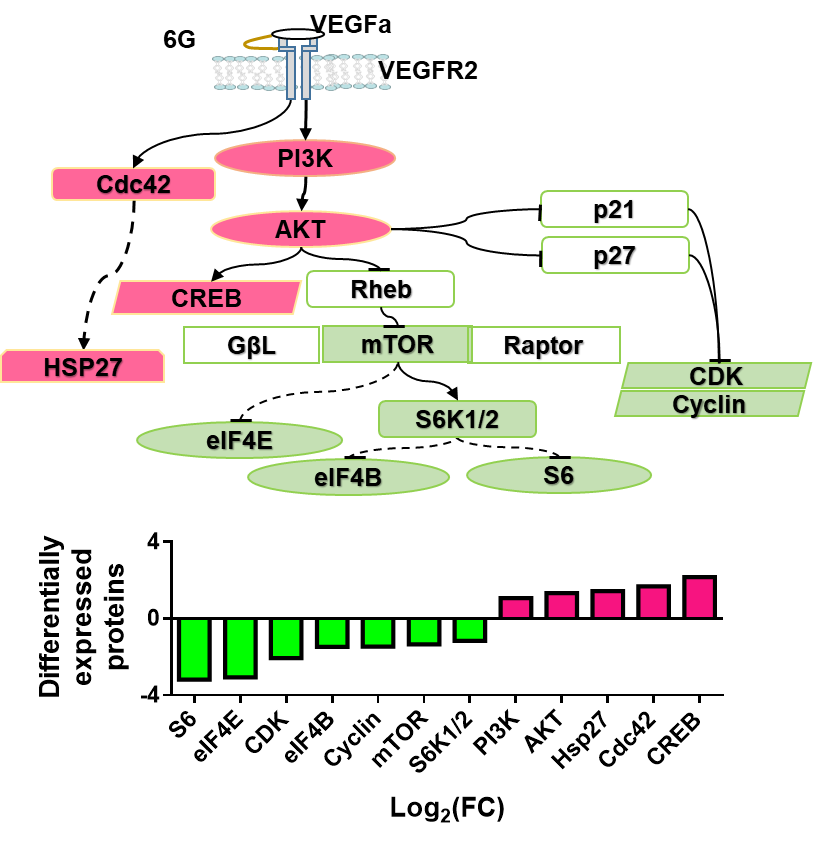


**Figure S2.** Pathway analysis revealed that 6G influenced VEGFa/VEGFR2 pathways.


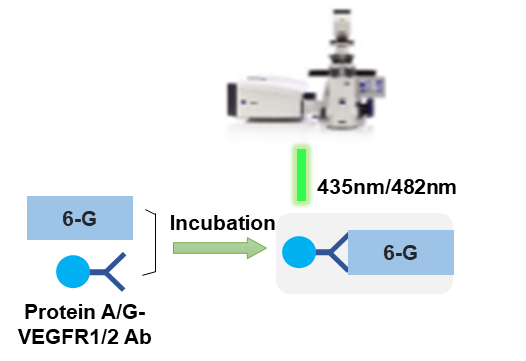


**Figure S3.** Positive pull-down assay of 6G with VEGFR1 and VEGFR2.


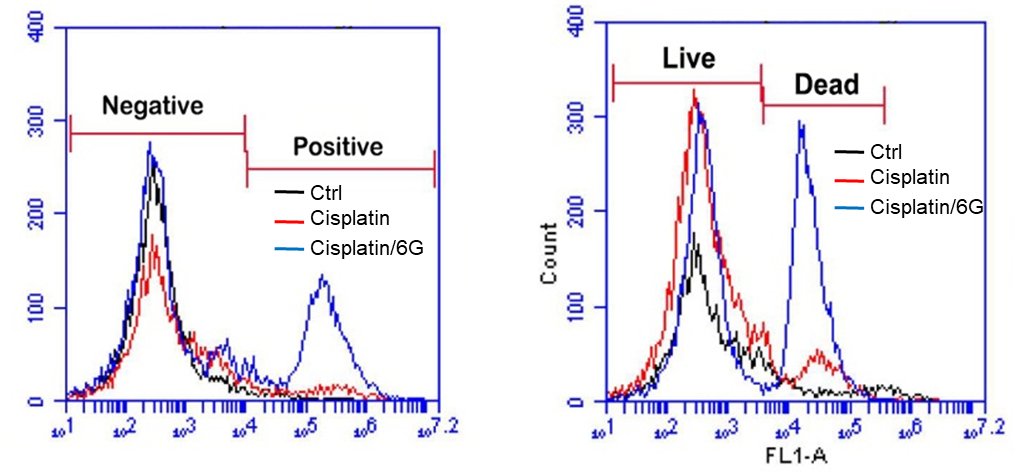


**Figure S4.** Flow cytometry analysis of 6G treated and untreated cells. Results showed that ROS levels and dead cells were significantly increased in the 6-gingerol and cisplatin co-treatment group.
